# Development of image-based decision support systems utilizing information extracted from radiological free-text report databases with text-based transformers

**DOI:** 10.1007/s00330-023-10373-0

**Published:** 2023-11-07

**Authors:** Sebastian Nowak, Helen Schneider, Yannik C. Layer, Maike Theis, David Biesner, Wolfgang Block, Benjamin Wulff, Ulrike I. Attenberger, Rafet Sifa, Alois M. Sprinkart

**Affiliations:** 1 Department of Diagnostic and Interventional Radiology, University Hospital Bonn, Bonn, Germany; 2 Fraunhofer Institute for Intelligent Analysis and Information Systems IAIS, Sankt Augustin, Germany

**Keywords:** Radiology, Deep learning, Intensive care units, Thorax

## Abstract

**Objectives:**

To investigate the potential and limitations of utilizing transformer-based report annotation for on-site development of image-based diagnostic decision support systems (DDSS).

**Methods:**

The study included 88,353 chest X-rays from 19,581 intensive care unit (ICU) patients. To label the presence of six typical findings in 17,041 images, the corresponding free-text reports of the attending radiologists were assessed by medical research assistants (“gold labels”). Automatically generated “silver” labels were extracted for all reports by transformer models trained on gold labels. To investigate the benefit of such silver labels, the image-based models were trained using three approaches: with gold labels only (M_G_), with silver labels first, then with gold labels (M_S/G_), and with silver and gold labels together (M_S+G_). To investigate the influence of invested annotation effort, the experiments were repeated with different numbers (*N*) of gold-annotated reports for training the transformer and image-based models and tested on 2099 gold-annotated images. Significant differences in macro-averaged area under the receiver operating characteristic curve (AUC) were assessed by non-overlapping 95% confidence intervals.

**Results:**

Utilizing transformer-based silver labels showed significantly higher macro-averaged AUC than training solely with gold labels (*N* = 1000: M_G_ 67.8 [66.0–69.6], M_S/G_ 77.9 [76.2–79.6]; *N* = 14,580: M_G_ 74.5 [72.8–76.2], M_S/G_ 80.9 [79.4–82.4]). Training with silver and gold labels together was beneficial using only 500 gold labels (M_S+G_ 76.4 [74.7–78.0], M_S/G_ 75.3 [73.5–77.0]).

**Conclusions:**

Transformer-based annotation has potential for unlocking free-text report databases for the development of image-based DDSS. However, on-site development of image-based DDSS could benefit from more sophisticated annotation pipelines including further information than a single radiological report.

**Clinical relevance statement:**

Leveraging clinical databases for on-site development of artificial intelligence (AI)–based diagnostic decision support systems by text-based transformers could promote the application of AI in clinical practice by circumventing highly regulated data exchanges with third parties.

**Key Points:**

*• The amount of data from a database that can be used to develop AI-assisted diagnostic decision systems is often limited by the need for time-consuming identification of pathologies by radiologists.*

*• The transformer-based structuring of free-text radiological reports shows potential to unlock corresponding image databases for on-site development of image-based diagnostic decision support systems.*

*• However, the quality of image annotations generated solely on the content of a single radiology report may be limited by potential inaccuracies and incompleteness of this report.*

**Supplementary Information:**

The online version contains supplementary material available at 10.1007/s00330-023-10373-0.

## Introduction

The application of AI-based DDSS has demonstrated the potential to increase efficiency and reading accuracy, thereby improving patient care [1–3]. The development of image-based DDSS requires a significant amount of training images for which it is known whether the disease of interest is present or not. If these annotations are not available for an image database, the number of images that can be used for DDSS development is limited by the need for time-consuming and costly image evaluation by annotators with considerable domain knowledge [1]. A further challenge is that medical data is subject to strict privacy regulations in most countries, making it difficult to share medical images for creating large international databases [4]. As a result, there is potential for local development of image-based DDSS in radiology clinics, as no data exchange in compliance with privacy regulations is required and diagnoses and findings are already made by radiology experts during clinical routine and documented in radiology reports.

These reports are commonly in free-text format, as many clinics have not integrated structured reporting into their daily routine [5]. To retrospectively identify a cohort of patients with a disease of interest from a report database, and thereby create labels for image-based DDSS development, it is necessary to assess the content of the reports in a fixed set of labels. Although the time-consuming and expert knowledge requiring reporting of images does not have to be repeated, retrospective assessment of the content of thousands of radiological reports to identify patient cohorts continues to involve considerable effort. To overcome this burden, various labeling and model pre-training strategies have been proposed to develop state-of-the-art transformer-based natural language processing (NLP) methods to classify the content of single radiological reports that can be used for retrospective structuring of chest X-ray report databases [6–8]. In a recent study, we investigated the potential of these different approaches for retrospective structuring of chest X-ray reports of ICU patients with respect to initial human annotation time required for subsequent NLP developments [9].

The results of a recent conference paper, in which the authors used X-ray images and English reports from the CheXpert dataset, indicate an advantage of transformers over rule-based systems in creating report content annotations for training image-based DDSS [10]. In another study using in-house chest X-ray examinations from a German university hospital, transformer-based annotations were also successfully used to develop image-based models [2]. Although manual report content was captured in “gold labels” for performance evaluations in these studies, the image-based DDSS were primarily trained with automatically generated “silver labels” from transformers. However, when a clinic develops a transformer to classify report content for on-site database structuring, manual annotations are typically performed. These are then also available as gold labels for subsequent training of the image-based DDSS. Therefore, in a realistic scenario, the development of transformer models has to be considered together with the subsequent development of image models.

The aim of this exploratory study is to gain insight into the potential and limitations of using manually created gold labels and transformer-based silver annotations of the contents of radiological reports for subsequent on-site development of image-based AI models for DDSS, also with respect to manual report annotation effort.

## Material and methods

### Overview

**Fig. 1:**
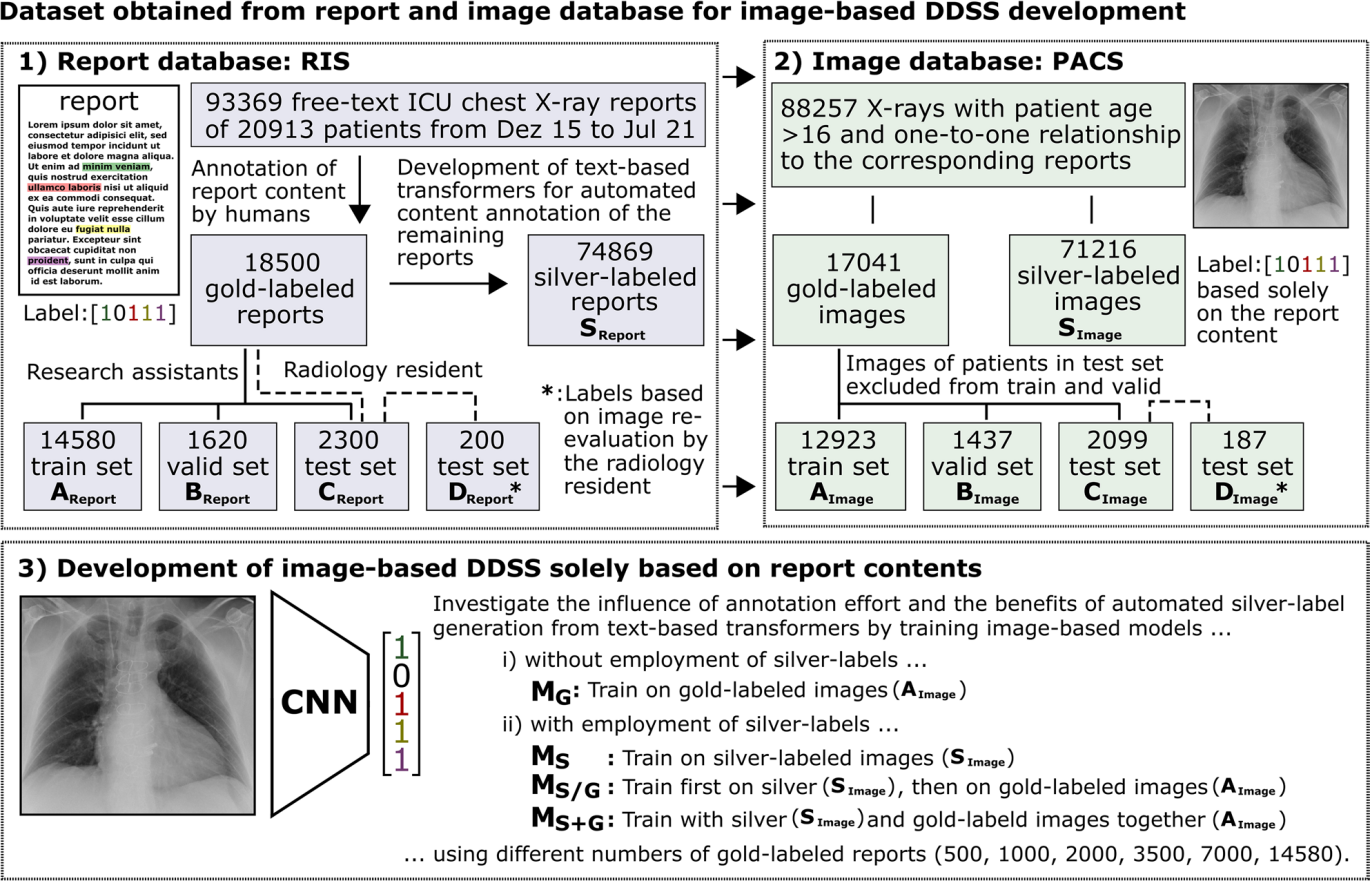
Overview of the entire study. (1) Report contents of chest X-ray examinations from intensive care unit (ICU) patients were exported from the radiology information system (RIS). For a portion of the exported reports, the text content was manually annotated (“gold labels”) and divided into a training (A_Report_), validation (B_Report_), and test (C_Report_) subset. Text-based transformer models that automatically “silver label” the content of the remaining reports were developed using the gold-labeled reports (S_Report_). The report annotation and development of the transformers shown in (1) was conducted in a previous study. For the current study, the corresponding images of 200 reports of the C_Report_ subset were re-evaluated to create image-based gold labels for testing and to assess the disagreement with the report content (D_Report_). (2) Images of patients older than 16 years with a clear one-to-one relationship to their associated report were exported from the Picture Archiving and Communication System (PACS). Consequently, the corresponding images to the different report datasets were available that have report content-based gold or silver labels (A_Image_, B_Image_, C_Image_, S_Image_) or image-based gold labels (D_Image_). (3) These datasets were used to explore different approaches for leveraging report content for the development of image-based DDSS

Radiological report content annotations generated in a previous study on transformer-based structuring of free-text radiology databases were used to label the corresponding ICU chest X-ray images for the development of DDSS systems [9]. Figure 1 illustrates the overall concept of the study and provides an overview of the different data sources and datasets used, as well as an overview of the different experiments conducted.

### Dataset

With institutional review board approval (AZ 411/21), written informed consent was waived. Approved data processing took place based on the health data protection act North Rhine-Westphalia (GDSG NW) §6 (2) state law NRW. The initial cohort includes 93,368 chest X-ray examinations with reports in German language of 20,913 ICU patients of the University Hospital Bonn from December 2015 to July 2021. The chest X-ray examinations were requested from various ICUs of our clinic (24% from anesthesiological, 24% from cardio-surgical, 20% from surgical, 11% from cardiological, 8% from neurological, 7% from internal medicine, 3% from oncological, and 3% from pediatric ICUs). In a previous study, two trained medical research assistants manually annotated the content of 18,000 chest X-ray reports under the supervision of a radiology resident with a mean annotation time of 39.4 s per report [9]. In these manually assessed reports, common indications were “position of medical devices” (45%) or presence of “pleural infiltrates” (39%), “pneumothorax” (38%), “pleural effusion” (30%), and “congestion” (22%). Additional 500 reports were annotated by the radiology resident and independently by the trained medical research assistants to assess inter-reader variability (mean accuracy of agreement: 97.4% and 97.3%, mean Cohen’s kappa: 0.92 and 0.91) [9]. These manually generated annotations are referred to as “gold labels.” This gold-labeled data set was randomly split into 14,580 training (A_Report_), 1620 validation (B_Report_), and 2300 hold-out test reports (C_Report_). The test set includes the 500 annotations from the radiology resident. For 200 reports of the test set that were annotated by the medical research assistants, the radiology resident reinterpreted imaging to assess overall label quality and to serve as an additional image labeled test set (D_Report_). In addition to these gold-labeled reports, automatically generated “silver labels” (S_Report_) were created by text-based transformer models (see Fig. 1). Detailed information about the annotation process can be found in supplement S5 and details on the development of the employed NLP algorithms can be found in the previous open-access study [9].

In 91,461 out of 93,368 examinations, a DICOM query of the picture archiving and communication system of the clinic returned only a single image object for the accession number associated with the report. Based on the unique one-to-one relationship between the report and the image, automatic export of the relevant image was performed while the remaining studies were excluded. Subsequently, patients younger than 16 years of age were excluded since the proportion and anatomy of not full-grown patients is different from that of full-grown patients. This resulted in a dataset with 88,257 images, 17,041 with gold labels and 71,216 with silver labels (S_Image_). No images were excluded due to quality aspects so that the data set reflects a realistic representation of clinical routine images. Furthermore, it was ensured that no images from other examination days of a patient from the test and validation cohort were in the training set. If there were several images of a patient acquired on different examination days within the test or validation cohort, one image was randomly selected. This resulted in a total of 12,923 training (A_Image_), 1437 validation (B_Image_), and 2099 test (C_Image_) images that had corresponding gold-labeled reports and 187 images from the test set with image-based gold labels (D_Image_). Based on these silver and gold annotated images, DDSS models were developed for the detection of pulmonary infiltrates, pleural effusion, pulmonary congestion, pneumothorax, and misplaced position of the central venous catheter (CVC).

### Pre-processing

An algorithm was applied to perform a rectangular crop of image areas outside the radiation field that were caused by acquiring the image with portable X-ray equipment in supine position. Details can be found in supplement S1. The cropped images were resized to 512 × 512 pixels. Then, a standard U-Net model segmented the lung to allow for computation of mean and variance within the lung mask for *z*-score normalization of the image values [11]. More information on the development of the lung segmentation U-Net used for pre-processing can be found in supplement S2. During training of the DDSS models, image augmentation methods were applied, which are described in detail in supplement S3. During training, all classes were up-sampled to at least 20% to avoid class imbalance in multi-label classification.

### Experiments

A DenseNet-121 Convolutional Neural Network with ImageNet pre-trained weights from the PyTorch torchvision library was used as established model for processing lung diseases in chest X-rays [12, 13]. To investigate the benefits of automatically transformer-generated silver labels, the model was trained with four approaches: (i) with gold labels only (M_G_), (ii) with silver labels only (M_S_), (iii) first with silver then with gold labels (M_S/G_), and (iv) with silver and gold labels together (M_S+G_).

To investigate these approaches with respect to different amounts of invested human annotation effort in an end-to-end manner, the development of transformers for silver label generation and the development of image-based DDSS using approaches i, ii, iii, and iv were repeated using different amounts of gold-labeled reports (*N*: 500, 1000, 2000, 3500, 7000, 14,580).

Binary cross entropy loss, AdamW optimizer, a one cycle learning rate schedule with a maximum learning rate of 0.01, a weight decay of 0.01, and a batch size of 128 was used for training [14]. While fine-tuning the M_S/G_ model on gold labels after training with silver labels, the maximum learning rate was reduced by a factor of 10^−1^ per dense block from the last to the first block, as commonly done when applying pre-trained weights [15, 16]. Detailed information on model architecture and training can be found in supplement S4. Model performance was assessed by single and macro-averaged AUC with 95% confidence intervals calculated by bootstrapping with 1000 resamples using torchmetrics v0.10.3. Non-overlapping CIs are interpreted as significant differences [17].

The report content classifying Bidirectional Encoder Representations from Transformers (BERT) models was developed in a previous study by pre-training the transformer with the unsupervised learning technique “masked language modeling” and subsequent fine tuning to gold-labeled reports [9]. Detailed information on the training and hyperparameters used can be found in the previous open-access study on on-site development of transformers in radiological clinics [9].

## Results

The main findings of the results are the following:

- The use of transformer-based silver labels is beneficial for the development of image-based DDSS of ICU chest X-ray examinations.
- Separated training with silver and then gold labels is advantageous if more than 2000 gold labels are available.
- There are differences between labels based on report content and labels based on image reinterpretation.

Table 1 shows the number of positive cases for the different pathological findings for all datasets used. The three classes with the lowest number of positive cases in the gold label dataset were pneumothorax (429), misplaced CVC (1071), and infiltrates (2560), and the two classes with the highest number of positive cases were congestion (4423) and effusion (6063).

**Table 1:** Number of positive cases for all silver- and gold-labeled training images (S_Image_, A_Image_) and the gold-labeled validation (B_Image_) and test subsets (C_Image_, D_Image_) used in this study. To investigate the influence of human annotation effort, the experiments were repeated with subsets of the gold-labeled training set A_Image_ with different numbers (*N*) of images

Datasets	S_Image_	A_Image_						B_Image_	C_Image_	D_Image_
Label type	Silver	Gold								
Purpose	Training	Training with various *N* of gold-labels						Valid	Test	Test
Number of images	56,797	12,923	6206	3096	1773	877	450	1437	2099	187
Findings	Number of positive cases in dataset splits									
Misplaced CVC	3766	1071	504	253	154	70	37	108	180	44
Effusion	36,922	6063	2868	1428	798	396	200	680	1004	113
Infiltrates	22,291	2560	1226	619	369	192	111	301	729	103
Congestion	17,360	4423	2105	1090	625	292	151	500	424	54
Pneumothorax	2450	429	210	122	71	34	15	51	74	34

**Fig. 2:**
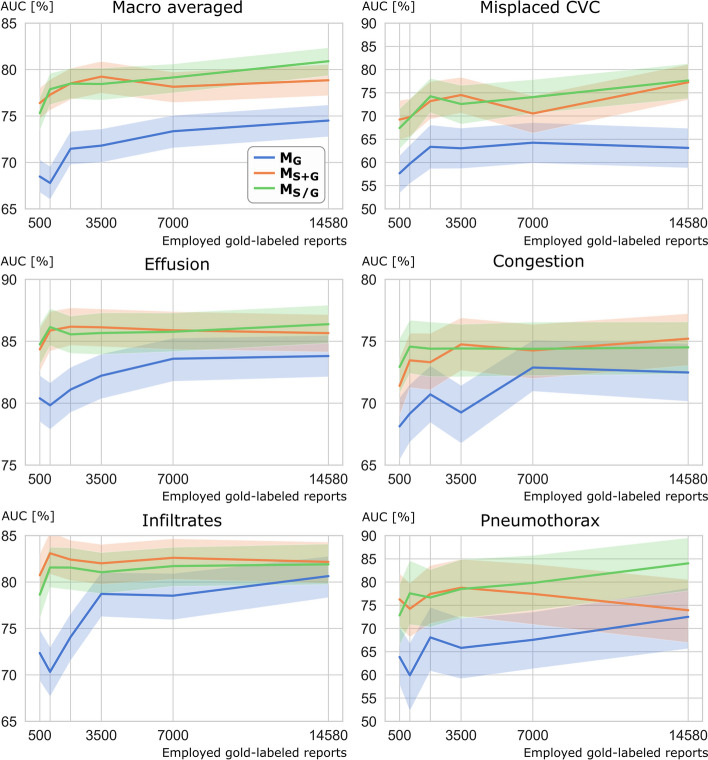
Area under the receiver operating characteristic curve (AUC) of the image-based DenseNet models for various levels of human annotation effort, represented as different numbers of employed manually labeled reports on the *x*-axis. Note that the transformer models for report content classification (silver labels generation) were also employing the same varying amounts of manually gold-labeled reports so that the end-to-end effect of different amounts of human annotation effort can be assessed. CVC, central venous catheter; M_G_: model trained on solely report-based gold labels; M_S+G_: model trained on report-based silver and gold labels together; M_S/G_: model trained first on report-based silver labels, then on gold labels

Table 2 and Fig. 2 show the diagnostic performance of the examined DDSS models evaluated on the test images with report-based labels for all classes and various numbers of gold-labeled reports. For all subsets with 1000 or more of gold-labeled reports employed, significantly higher macro-averaged and misplaced CVC AUC scores were observed for the DDSS models employing transformer generated silver labels (M_S_, M_S+G_, and M_S/G_) compared to the DDSS model trained solely on gold-labeled images (M_G_). For pleural effusion, M_S_, M_S+G_, and M_S/G_ performed significantly better than M_G_ when 3500 or a lower number of gold-labeled reports were available. The same observation was made for pulmonary infiltrates when only 2000 or fewer gold-labeled reports were available. M_S+G_ performed better than M_S_ and M_S/G_ when using only 500 gold-labeled reports for the three findings pneumothorax, misplaced CVC, and pulmonary infiltrates, which had the lowest number of positive cases. Table 2 additionally lists the diagnostic performance on the test data set with image-based labels (D_Image_). It was observed that for macro-average, misplaced CVC AUC, M_S+G_ had higher values than M_S_ and M_S/G_ when 2000 or fewer gold-labeled reports were available and M_S/G_ had higher values than M_S_ and M_S+G_ when more than 2000 gold-labeled reports were used.

**Table 2:** Area under the receiver operating characteristic curve (AUC) in % observed for the hold-out test set of 2099 images that were labeled by report content and for the hold-out test set of 187 images that were labeled by re-evaluating imaging. The image-based models were trained on report-based labels with four different approaches: solely on gold labels (M_G_), solely on silver labels (M_S_), first with silver, then with gold labels (M_S/G_) and with silver and gold labels together (M_S+G_). The transformer and image-based models were trained with various numbers (*N*) of gold-labeled reports and images to investigate the influence of annotation effort on DDSS model performance. For M_S_, solely silver-labeled images were used generated by the transformer trained with *N* gold labels. The highest performances of the models trained with the same number of gold labels are indicated by bold font for both test sets. Significant differences between the AUCs of M_G_ and M_S_ or M_G_ and M_S+G_ or M_G_ and M_S/G_ are indicated by * and between the AUCs of the same model (M_G_/M_S_/M_S+G_/M_S/G_) tested on report- or image-based labels with †

Number of gold labels used		Test-set labeled by report content (*N* = 2099)								Test-set labeled by image content (*N* = 187)							
		M_G_	M_S_	M_S+G_	M_S/G_	M_G_	M_S_	M_S+G_	M_S/G_	M_G_	M_S_	M_S+G_	M_S/G_	M_G_	M_S_	M_S+G_	M_S/G_
Reports	Images	AUC macro-averaged				Misplaced CVC				AUC macro-averaged				Misplaced CVC			
14,580	12,935	74.5	79.7*	78.8*	80.9*	63.1	73.5*	77.3*	77.7*	75.8	84.6*	82.4	84.8*	61.3	81.8*	79.3*	83.4*
7000	6206	73.4	78.1*	78.2*	79.2*	64.3	73.4*	70.5	74.1*	76.5	82.1*	82.0	**82.8**	68.8	76.4	73.6	**76.7**
3500	3096	71.8	78.3*	79.2*	78.5*	63.1	71.9*	74.5*	72.6*	75.7	82.9*	81.8	83.0*	65.4	77.7	73.1	**77.9**
2000	1773	71.5	77.4*	78.5*	78.5*	63.4	71.3*	73.2*	74.3*	73.5	79.9	81.5*	81.1*	67.4	71.7	**75.9**	75.6
1000	877	67.8	77.5*	77.3*	77.9*	59.7	68.6*	69.8*	69.6*	69.5	80.3*	82.8*†	80.2*	57.5	69.9	**76.0**	69.5
500	450	68.5	75.1*	76.4*	75.3*	57.7	65.7	69.2*	67.4*	68.9	78.9*	80.1*	76.9*	58.9	72.5	**76.7**	69.7
Reports	Images	Pleural effusion				Pulmonary congestion				Pleural Effusion				Pulmonary congestion			
14,580	12,935	83.8	86.1	85.7	**86.4**	72.5	73.5	**75.2**	74.5	84.5	87.9	**88.6**	87.5	81.1	81.7	**84.8**†	83.9†
7000	6206	83.6	84.5	**85.9**	85.8	72.9	74.2	74.3	**74.4**	84.1	85.5	**87.7**	86.6	81.9†	84.8†	84.3†	**84.8**†
3500	3096	82.2	85.7*	86.1*	85.7*	69.3	74.4*	74.8*	74.4*	82.2	88.2	87.1	**88.5**	81.9†	83.4†	83.0†	**83.9**†
2000	1773	81.1	85.8*	86.2*	85.6*	70.7	73.9	73.3	**74.4**	81.3	86.7	**87.8**	87.6	80.6†	82.3†	82.2†	**83.5**†
1000	877	79.8	86.3*	85.9*	86.2*	69.2	74.3*	73.5	74.6*	79.1	**87.2**	86.8	86.8	81.6†	83.8†	**84.3**†	83.9†
500	450	80.4	84.4*	84.4*	84.8*	68.1	72.7	71.4	72.9*	79.4	85.5	82.1	**86.3**	76.5	**85.4**†	81.8†	85.0†
Reports	Images	Pulmonary infiltrates				Pneumothorax				Pulmonary infiltrates				Pneumothorax			
14,580	12,935	80.6	82.3	**82.2**	81.9	72.5	83.4	73.9	**84.0**	73.3	**81.3**	79.1	77.3	79.1	90.3	80.2	**91.9**
7000	6206	78.5	81.4	**82.6**	81.7	67.6	77.2	77.5	**79.8**	76.3	**79.4**	79.2	77.8	71.2	84.7	85.2	88.0*
3500	3096	78.7	81.2	**82.0**	81.1	65.8	78.6	78.8*	78.5	78.7	76.1	**77.5**	76.0	70.2	89.2*	88.0*	88.8*
2000	1773	74.1	81.8*	82.4*	81.6*	68.1	74.0	**77.4**	76.7	68.2	**79.4**	77.5	77.8	69.9	79.1	**83.8**	81.1
1000	877	70.3	80.9*	83.1*	81.6*	59.9	77.5*	74.2*	77.6*	63.6	74.5	81.3*	75.6	65.9	86.3*	85.5*	85.3*
500	450	72.4	79.1*	80.7*	78.6*	63.9	73.6	76.3*	72.8	69.0	**75.8**	74.8	72.2	60.8	75.3	84.9*	71.4

Interestingly, the macro-averaged AUC of the models evaluated on the test set with image-based labels were higher than the macro-averaged AUC of the same models evaluated on the report-based labeled test set. For pulmonary congestion, AUC values of all M_S+G_ and M_S/G_ models evaluated on the dataset with image-based labels were significantly higher than the same models tested on the report-based labels. Detailed metrics for M_S/G_ for which the highest macro-averaged AUC values were observed in both the report- and image-labeled test sets can be found in Table 3.

**Table 3:** Detailed metrics for the receiver operating characteristic analysis of the best model M_S/G_ trained with all available data on both test sets with report and image-based labels. The area under the receiver operating characteristic curve (AUC) in % is given per class. Also, sensitivity and specificity in % are given per class for binary classifications. Thresholds were calculated by the Youden-Index on the training set and applied to the test set

	Test-set labeled by report content			Test-set labeled by image content		
Classes	AUC	Sensitivity	Specificity	AUC	Sensitivity	Specificity
Misplaced CVC	77.6	64.4	74.1	83.4	70.5	76.9
Pleural Effusion	86.4	71.9	83.3	87.5	73.5	83.8
Pulmonary Congestion	74.5	57.8	75.9	83.9	60.2	88.1
Pulmonary Infiltrates	81.9	78.3	70.7	77.3	77.8	66.9
Pneumothorax	84.0	87.8	60.6	91.8	97.1	60.1
Overall	80.9	72.0	72.9	84.8	75.8	75.2

Table 4 shows the agreement between the labels based on report content and the labels based on image re-assessment of the gold-labeled test set (D_Image_). When comparing report content annotation with image re-evaluation, the lowest AUC (93.5%, 95.5%) and accuracy values (93.0%, 93.6%) were observed for pulmonary infiltrates and congestion. For pulmonary infiltrates, sensitivity was 100% and specificity 91.0%, and for pulmonary congestion, sensitivity was 89.3% and specificity 97.6%.

**Table 4:** Accuracy, sensitivity, specificity, area under the receiver operating characteristic curve (AUC), precision, and F1-Score between report-based generated labels from medical research assistants and image-based labels from a radiology resident. A total of 187 images were considered during the evaluation

Class	Accuracy	Sensitivity	Specificity	AUC	Precision	F1-score
Misplaced CVC	97.9	100.0	97.2	98.6	91.7	95.7
Pleural effusion	95.7	95.6	95.9	95.8	97.3	96.4
Pulmonary Congestion	93.0	89.3	97.6	93.5	97.9	93.4
Pulmonary infiltrates	93.6	100.0	91.0	95.5	81.8	90.0
Pneumothorax	98.9	100.0	98.7	99.3	94.4	97.1
Overall	95.8	97.0	96.1	96.5	92.6	97.0

## Discussion

In this study, we investigated the potential and limitations of extracting findings from radiology reports, also employing text-based transformers, to annotate the corresponding images for on-site development of image-based DDSS. In many countries, such as Germany, data protection regulations strictly restrict the exchange of radiological reports and images that contain personal data closely linked to sensitive medical information with third parties (e.g., AI companies). The opportunity to develop these systems using unstructured, retrospectively collected data on-site in radiology clinics could drive the development and ultimately the application of specialized AI models in routine clinical practice. These AI applications could, for example, provide an initial assessment immediately after image acquisition by the technical assistants and therefore could contribute to faster detection and treatment of emergencies.

For the following reasons, we considered ICU chest X-ray examinations suitable for investigating this subject. With ICU chest X-ray examinations, there is usually a clear one-to-one relationship between the report and the image, without the report describing multiple images of an imaging series. The image data is two-dimensional, which makes the development of DDSS less complex. The images of ICU patients frequently present severe pathologies, which reduces class imbalance for training of DDSS. Lastly, rapid identification of pathologies is essential in these critically ill patients, which makes DDSS of high interest [18]. However, ICU chest X-ray examinations are in principle more demanding to analyze than regular chest X-rays. One reason for this is that ICU patients suffer from a variety of serious conditions and may receive a variety of treatments. ICU patients may be mechanically ventilated; there may be tubes, catheters, and other medical devices that can alter, obscure, or distort the anatomy of the lungs. Another reason is a frequently limited image quality. ICU X-rays of critically ill patients are typically acquired with portable X-ray scanners in lying position, which can induce gravity related alterations in location and appearance of organs and tissues. Also, the condition of the patient and the medical equipment may not allow ICU patients to be positioned accurately perpendicular to the X-ray beam resulting in further image distortion.

Despite these particular challenges, the image-based model utilizing both manual and transformer-based report content labels showed a macro-average AUC of 84.8% on the image-labeled test set. This indicates the potential of transformers for unlocking the content of free-text reports of radiological report databases to ultimately develop image-based DDSS without the need for image re-evaluations. The investigation of the performance of the models developed with different numbers of gold-labeled reports demonstrated that it is beneficial to train with silver and gold labels together when only 2000 or fewer reports have been annotated by humans. If more reports can be annotated, separated training with silver and then gold labels appeared preferable in our study compared to training with a mixture of gold- and silver-labeled images. This is in line with the observation on the two test datasets that the model trained with only silver-labeled images performed better than the model trained with mixed label types when 14,580 gold-labeled reports were available to train the silver label generating transformer.

In addition to the report-based labeled test set, we also generated an image-based labeled test to investigate discrepancies between report content and image findings that potentially pose a limitation to the use of manual and transformer generated report-based labels for on-site DDSS development. Interestingly, it was observed that all models demonstrate higher macro-averaged AUC values when evaluated on the test set with image-based labels compared to evaluation of the same models on the report-based labeled test set. A previous conference paper already discussed potential reasons that can lead to discrepancies between report-content and image findings [19]:

i) Findings that are not of high relevance to the current clinical condition of the patient might not be mentioned in the report, although they may be present within imaging.
ii) Findings within a report may be based on information that is not content of the report, e.g., information from reports from previous examinations or clinical/laboratory parameters.
iii) Borderline image findings could yet be remarked by the attending radiologist for assurance and consequently be considered equally as definite findings for the DDSS training.
iv) And lastly, the radiologist might have made an error during the reporting. Also, further errors may occur during the subsequent annotation of the report content by the human annotators and/or by the transformers.

To assess the overall label discrepancies potentially caused by the above-listed reasons, the results of the image reassessments were compared with the gold labels based on the report content. This revealed high specificity combined with lower sensitivity for pulmonary congestion; i.e., congestions present within imaging were occasionally not mentioned in the report. However, it was rare that the image reader disagreed after re-evaluation when the pathology was mentioned in the report. One could speculate that minor congestions that were not of major importance for the current clinical question were occasionally not reported, as also described in above-described scenario i. Interestingly, both models pre-trained with silver labels showed significantly higher AUC values for pulmonary congestions when evaluated on the test subset with image-based labels compared to the test subset with report-based labels. This indicates that despite the observed limited sensitivity of the report content for pulmonary congestion, the DDSS models learned to correctly detect the pathology also in some cases where it was not mentioned in the corresponding reports of the test subjects.

For pulmonary infiltrates, high sensitivity with lower specificity was observed when comparing report content with image re-evaluation. This implies that the reader who re-assessed infiltrates solely on imaging occasionally disagreed with the occurrence of the pathology in the report. However, when the image reader identified infiltrates, this consistently agreed with the report content.

The more frequent recognition of infiltrates in the report texts compared a to re-evaluation of the images may result from additional information available to the attending radiologist at the time of reporting, but which is not content of the report text, as described in scenario ii. For example, recent inflammatory laboratory values and results of previous clinical examinations or previous radiological reports may have encouraged the examiner to describe a lesion as an infiltrate. The more frequent inclusion of infiltrates in the report texts may also be caused by the difficulty identifying a lesion as pulmonary infiltrate on ICU images with patients in lying position. This may increase the number of borderline cases that could still be mentioned in the report by the attending radiologist, as described above in scenario iii.

Other work propose the following approaches to address this challenge of imprecise direct mapping of report and image content. Similar to the current study, one study proposes to first train an image-based deep learning model with labels that are derived from the content of the corresponding reports [19]. The authors claim that the class probabilities provided by this image-based model are more precise labels for the development of text-based transformers in comparison to the initial labels derived from the report content. A follow-up study shows that the labels of this improved transformer also lead to higher performance of the image-based DDSS [10]. Another paper proposes a more sophisticated approach for the annotation of chest X-ray images based on report content by also assessing a second report of a recent CT scan [2]. If the contents of both reports agree, the authors assume that the X-ray report text is accurate. To reduce noise in the dataset caused by imprecise report texts, the authors also propose to first train an image-based model on the noisy data. Then, some image-based labels are manually created by reviewing cases for which the prediction of this model strongly disagrees with the report content label. This more sophisticated approach, involving annotation of two reports and reviewing of imaging, showed promising results in improving the quality of the labels. However, the scope of eligible patients is limited, as imaging and reporting must be available for both modalities and the manual re-evaluation of images requires costly time of radiological experts. Other work presented algorithmic approaches to increase robustness to noisy labels during training of an image-based deep learning model. For example, one paper proposes to extend the loss function to allow the model to ignore cases during training that are strong outliers due to inaccurate labels [20]. This warrants further studies investigating the utility of more time-consuming labeling approaches versus the use of algorithmic approaches to handle the noise of labels extracted directly from report contents for on-site DDSS development in radiology departments.

The use of transformer-based report content annotation for DDSS developments has a further limitation that is not apparent from the study results. Unlike the ICU chest X-ray examination used in this study, the report content of, e.g., MRI examinations are based on multiple imaging sequences. Therefore, further considerations are required when applying the concept to other imaging modalities.

## Conclusion

The results show that report content extraction by transformers could aid in unlocking unstructured retrospective routine data in radiological clinics for on-site DDSS development. However, noisy labels caused by imperfect report and image content mapping pose challenges to the presented approach. Therefore, on-site development of image-based DDSS could potentially benefit from more sophisticated annotation pipelines that include information beyond the corresponding radiological report and from algorithmic approaches to handle noisy labels. Moreover, the application of the approach of employing report contents for training of image-based DDSS should be further investigated for imaging examinations where the report is based on multiple images.

### Supplementary Information

Below is the link to the electronic supplementary material.

Supplementary file1 (PDF 299 KB)

## Abbreviations

AUC: Area under the receiver operating characteristic curve
AI: Artificial intelligence
CVC: Central venous catheter
DDSS: Diagnostic decision support systems
ICU: Intensive care unit
NLP: Natural language processing

## Declarations

### Guarantor

The scientific guarantor of this publication is PD Dr.-Ing. Alois Martin Sprinkart.

### Statistics and biometry

No complex statistical methods were necessary for this paper.

### Study subjects or cohorts overlap

The present study is a follow-up to a study published in European Radiology on the transformer-based structuring of free-text radiology databases (Nowak, S., Biesner, D., Layer, Y.C. et al Eur Radiol (2023). https://doi.org/10.1007/s00330-023-09526-y). The manual and transformer-based report annotations generated in the previous study were used in the current study to annotate the corresponding ICU radiographs to investigate the value of these report-based annotations for the development of image-based DDSS systems.

### Methodology

• retrospective

• experimental study

• performed at one institution

## References

[1] Hosny A, Parmar C, Quackenbush J, Schwartz LH, Aerts HJ (2018). Artificial intelligence in radiology. Nat Rev Cancer.

[2] Niehues SM, Adams LC, Gaudin RA (2021). Deep-learning-based diagnosis of bedside chest X-ray in intensive care and emergency medicine. Invest Radiol.

[3] Mango VL, Sun M, Wynn RT, Ha R (2020). Should we ignore, follow, or biopsy? Impact of artificial intelligence decision support on breast ultrasound lesion assessment. AJR Am J Roentgenol.

[4] Richter-Pechanski P, Amr A, Katus HA, Dieterich C (2019). Deep learning approaches outperform conventional strategies in de-identification of German medical reports. Stud Health Technol Inform.

[5] Nobel JM, Kok EM, Robben SG (2020). Redefining the structure of structured reporting in radiology. Insights Imaging.

[6] Smit A, Jain S, Rajpurkar P, Pareek A, Ng AY, Lungren MP (2020) CheXbert: combining automatic labelers and expert annotations for accurate radiology report labeling using BERT. arXiv preprint arXiv:2004.09167

[7] McDermott MB, Hsu TMH, Wenig WH, Ghassemi M, Szolovits P (2020). Chexpert++: Approximating the chexpert labeler for speed, differentiability, and probabilistic output. Proceedings of PMLR.

[8] Bressem KK, Adams LC, Gaudin RA (2020). Highly accurate classification of chest radiographic reports using a deep learning natural language model pre-trained on 3.8 million text reports. Bioinformatics.

[9] Nowak S, Biesner D, Layer YC (2023). Transformer-based structuring of free-text radiology report databases. Eur Radiol.

[10] Jain S, Smit A, Ng AY, Rajpurkar P (2021) Effect of radiology report labeler quality on deep learning models for chest X-ray interpretation. arXiv preprint arXiv:2104.00793

[11] Ronneberger O, Fischer P, Brox T (2015) U-net: convolutional networks for biomedical image segmentation. In proceedings of MICCAI 2015 18:234-241

[12] Huang G, Liu Z, Van Der Maaten L, Weinberger KQ (2017) Densely connected convolutional networks. In proceedings of CVPR 2017 4700-4708

[13] Irvin J, Rajpurkar P, Ko M (2019). Chexpert: a large chest radiograph dataset with uncertainty labels and expert comparison. Proc AAAI Conf Artif Intell.

[14] Smith LN (2018) A disciplined approach to neural network hyper-parameters: Part 1 -- learning rate, batch size, momentum, and weight decay. arXiv preprint arXiv:1803.09820

[15] Nowak S, Mesropyan N, Faron A (2021). Detection of liver cirrhosis in standard T2-weighted MRI using deep transfer learning. Eur Radiol.

[16] Luetkens JA, Nowak S, Mesropyan N (2022). Deep learning supports the differentiation of alcoholic and other-than-alcoholic cirrhosis based on MRI. Sci Rep.

[17] Cumming G (2009). Inference by eye: Reading the overlap of independent confidence intervals. Stat Med.

[18] Spiritoso R, Padley S, Singh S (2015). Chest X-ray interpretation in UK intensive care units: A survey 2014. J Intensive Care Soc.

[19] Jain S, Smit A, Truong SQ et al (2021) VisualCheXbert: addressing the discrepancy between radiology report labels and image labels. In Proceedings of CHIL 2021 105-115

[20] Thulasidasan S, Bhattacharya T, Bilmes J, Chennupati G, Mohd-Yusof J (2019) Combating label noise in deep learning using abstention. arXiv preprint arXiv:1905.10964

