# Supplementary material for: Development of image-based decision support systems utilizing information extracted from radiological free-text report databases with text-based transformers
Source: Eur Radiol. 2023 Nov 7;34(5):2895–904. doi: 10.1007/s00330-023-10373-0 (PMC11126497; doi:10.1007/s00330-023-10373-0)
Supplement: Supplementary file 1 — Supplementary file1 (PDF 299 KB) [file 330_2023_10373_MOESM1_ESM.pdf]

Development of image-based decision support systems utilizing information extracted from radiological free-text report databases with text-based transformers

Electronic Supplementary Material (ESM)

Eur Radiol (2023) Nowak S, Schneider H, Layer YC et al

S1 Collimator Crop

The exposed area of a rectangular collimator is described by modeling each of the four edges as a linear function. Assuming that the exposure area features on average greater values than the outside, points are moved iteratively from the edge of the image into the image, so that the difference of average values behind and front of a point is maximized. Doing this for an entire edge of an image produces enough points that are laying on the respective collimator's edge so that applying a linear regression with Huber metric yields a good approximation of the collimator's edge.

S2 Segmentation U-Net

For training the segmentation model, 820 lung fields were annotated by research assistants under the guidance of a radiology resident. The data set was separated into training, validation, and test sets (70%/15%/15%). The trained model achieves a Dice Score of 96.27 on the hold-out test set.

S3 Data augmentation

To avoid overfitting, data augmentation methods were used during training. In detail, the settings of the RandomAffine Augmentation class of the torchvision transforms package were Rotation (-15, 15), Translation (0.05, 0.05), Scaling (0.95, 1.05), Shearing (-10, 10). Additionally, Gaussian noise with a mean of 0.1 and standard deviation of 0.08 were added to the images.

S4 Additional information on model training

The general concept of the DenseNet convolutional neural network architecture used in the current study is the combination of all feature maps of the previous convolutional layers by concatenation as input for each following convolutional layer. The goal is to strengthen feature propagation and the reuse of features, to minimize the problem of vanishing-gradients, while also reducing the number of trainable parameters [1]. For the experiments the DenseNet-121 version was used that featured 6958981 trainable parameters.

The models developed exclusively on gold labels (M_G) were trained for 1000 epochs, except for the model trained on all gold-labeled data, which was trained for 300 epochs. Early stopping was used, i.e. if no improvement in the macro-averaged area under the receiver operating curve score was observed on the validation data after 100 epochs, the training was terminated. All models that were trained with silver and gold labels together (M_{S+G}) were trained for 300 epochs with early stopping after 25 epochs of no improvement. All models that were first trained on silver and then on gold labels ($M_{S/G}$) were first trained for 300 epochs on silver labels with early stopping after 25 epochs of no improvement and then 100 epochs on gold labels.

S5 Detailed information on the report annotation process

Two medical research assistants under the supervision of a radiology resident and by application of the open-source software doccano [2] annotated the occurrence of 35 labels in the radiology reports that include not only findings but also further information, e.g., on the indication. The assignment was made by the context of the information in the free-text reports and not by individual words and in case of ambiguity, the annotators were instructed to consult the supervisor. All 35 labels that were annotated in the previous open-access study were [3]:

- *Identifying information*: Names, dates
- *Indications*:
  - pulmonary infiltrates
  - pleural effusion
  - pulmonary congestion
  - pneumothorax
  - material position
  - other
- *Findings*:
  - pulmonary infiltrates
  - pleural effusion
  - pulmonary congestion
  - pneumothorax
  - atelectasis
  - soft tissue emphysema
  - mediastinal emphysema
  - mediastinal shift
  - fracture
  - other extrathoracic secondary findings
- *Material positions* (regular/misplaced):
  - central venous catheter (CVC)
  - pleural drainage
  - endotracheal tube
  - tracheal cannula
  - gastric tube
  - cardiac pacemaker
  - other
- *Image quality*:
  - image truncated
  - image skewed

A subset of six findings that are frequently raised during a patient's ICU stay and that have significant clinical relevance were selected from the entire label set for the development of the text-based transformers and ultimately the image-based predictive models.

S6 Tables with Confidence intervals

Number of gold-labels used		Test-set labeled by report content (N=2099)								Test-set labeled by image content (N=187)							
		M_G	M_S	M_{S+G}	$M_{S/G}$	M_G	M_S	M_{S+G}	$M_{S/G}$	M_G	M_S	M_{S+G}	M_{SG}	M_G	M_S	M_{S+G}	$M_{S/G}$
reports	images	**AUC Macro-averaged**				**Misplaced CVC**				**AUC Macro-averaged**				**Misplaced CVC**			
14580	12935	74.51 [72.78-76.19]	79.75* [78.19-81.28]	78.85* [77.21-80.55]	80.9* [79.35-82.35]	63.13 [58.84-67.35]	73.45* [69.72-76.89]	77.26* [73.5-81.12]	77.65* [73.77-81.27]	75.84 [72.27-79.11]	84.58* [81.64-87.39]	82.39 [78.86-85.65]	84.79* [81.97-87.61]	61.25 [51.18-70.8]	81.77* [74.79-88.13]	79.26* [71.63-86.34]	83.42* [75.57-89.08]
7000	6206	73.36 [71.58-75.06]	78.14* [76.48-79.87]	78.15* [76.47-79.74]	79.15* [77.49-80.6]	64.25 [59.88-68.57]	73.39* [69.34-77.35]	70.54 [66.33-74.36]	74.07* [70.56-77.82]	76.46 [72.96-79.86]	82.14 [78.73-85.16]	82.01 [78.83-85.34]	82.79 [79.61-85.95]	68.77 [59.83-78.07]	76.37 [68.38-83.66]	73.62 [65.64-81.64]	76.65 [68.93-83.57]
3500	3096	71.81 [70.02-73.6]	78.34* [76.71-79.9]	79.24* [77.48-80.88]	78.45* [76.73-80.16]	63.05 [58.71-67.38]	71.91* [67.91-75.9]	74.52* [70.65-78.33]	72.59* [68.29-76.63]	75.67 [71.72-79.09]	82.91* [79.45-85.83]	81.75 [78.64-84.8]	83.0* [79.64-86.0]	65.37 [56.26-74.39]	77.67 [69.8-84.79]	73.12 [65.35-80.69]	77.88 [70.51-84.74]
2000	1773	71.47 [69.8-73.33]	77.45* [75.72-79.1]	78.51* [76.77-80.14]	78.49* [76.87-80.14]	63.36 [58.66-68.06]	71.25* [66.97-74.96]	73.21* [69.37-77.41]	74.25* [70.74-78.09]	73.46 [69.37-77.33]	79.86 [76.4-83.1]	81.47* [77.98-84.86]	81.12* [77.62-84.31]	67.37 [58.23-76.33]	71.73 [63.32-79.31]	75.94 [67.89-82.66]	75.6 [66.92-82.61]
1000	877	67.79 [66.04-69.58]	77.52* [75.68-79.14]	77.29* [75.65-78.93]	77.89* [76.22-79.59]	59.72 [55.5-63.74]	68.58* [64.51-72.57]	69.77* [65.58-73.7]	69.6* [65.26-73.64]	69.52 [65.81-73.22]	80.31* [76.7-83.65]	82.79*† [79.36-85.97]	80.21* [76.88-83.58]	57.47 [47.62-67.55]	69.88 [61.56-78.03]	75.99 [67.56-83.72]	69.52 [61.62-77.45]
500	450	68.48 [66.79-70.28]	75.09* [73.24-76.86]	76.4* [74.74-78.02]	75.31* [73.49-77.01]	57.66 [53.46-61.47]	65.73 [61.27-70.2]	69.24* [64.92-73.36]	67.41* [62.94-71.59]	68.92 [65.12-72.37]	78.88* [74.78-82.45]	80.06* [76.82-83.27]	76.91* [73.07-80.51]	58.92 [49.0-68.66]	72.50 [63.59-81.22]	76.73 [68.25-84.47]	69.69 [61.37-78.4]

reports	images	**Pleural Effusion**				**Pulmonary Congestion**				**Pleural Effusion**				**Pulmonary Congestion**			
14580	12935	83.81 [82.14-85.48]	86.09 [84.54-87.61]	85.66 [84.14-87.15]	86.38 [84.85-87.91]	72.48 [70.16-74.82]	73.51 [71.22-75.64]	75.21 [73.05-77.23]	74.51 [72.35-76.57]	84.48 [77.85-89.91]	87.98 [82.77-92.13]	88.56 [83.87-92.7]	87.48 [82.55-91.93]	81.13 [74.21-87.26]	81.66 [75.24-87.11]	84.82† [79.41-90.33]	83.88† [78.34-89.21]
7000	6206	83.59 [81.78-85.25]	84.52 [82.91-86.16]	85.89 [84.35-87.4]	85.77 [84.17-87.29]	72.88 [70.97-75.09]	74.17 [71.89-76.26]	74.26 [71.99-76.36]	74.39 [72.23-76.55]	84.12 [78.44-89.65]	85.46 [79.06-90.88]	87.65 [82.57-92.55]	86.63 [81.53-91.79]	81.92† [75.33-87.78]	84.79† [79.24-89.89]	84.34† [78.57-89.88]	84.77† [79.08-89.59]
3500	3096	82.22 [80.37-83.97]	85.66* [84.1-87.15]	86.13* [84.58-87.61]	85.67* [83.94-87.3]	69.25 [66.78-71.43]	74.37* [72.11-76.43]	74.75* [72.65-76.89]	74.42* [72.14-76.38]	82.22 [75.8-88.16]	88.17 [83.18-92.6]	87.11 [82.08-91.73]	88.48 [83.44-92.83]	81.89† [75.47-87.62]	83.4† [773.96-88.61]	83.04† [76.89-88.8]	83.92† [77.98-89.16]
2000	1773	81.1 [79.27-82.9]	85.75* [84.11-87.24]	86.18* [84.68-87.7]	85.56* [84.04-87.04]	70.71 [68.45-73.01]	73.93 [71.85-76.08]	73.31 [71.08-75.64]	74.4 [72.14-76.55]	81.27 [74.46-87.16]	86.74 [81.13-91.7]	87.83 [82.68-92.57]	87.57 [82.28-92.65]	80.58† [73.95-86.09]	82.34† [76.34-87.86]	82.2† [76.1-87.72]	83.48† [77.44-89.02]
1000	877	79.83 [77.9-81.65]	86.32* [84.75-87.8]	85.87* [84.22-87.4]	86.15* [84.65-87.64]	69.16 [66.87-71.51]	74.33* [72.29-76.43]	73.46 [71.26-75.65]	74.57* [72.4-76.7]	79.14 [72.23-85.37]	87.18 [81.99-91.46]	86.82 [81.42-91.02]	86.79 [81.91-91.44]	81.56† [75.47-87.31]	83.77† [78.01-88.98]	84.3† [77.97-89.3]	83.86† [78.38-88.91]
500	450	80.39 [78.51-82.23]	84.38* [82.65-86.04]	84.35* [82.59-86.06]	84.75* [83.12-86.33]	68.13 [65.46-70.44]	72.71 [70.37-74.98]	71.4 [69.03-73.64]	72.93* [70.81-75.23]	79.41 [72.95-85.34]	85.46 [80.07-90.59]	82.12 [75.08-87.94]	86.25 [79.88-91.64]	76.51 [69.72-83.77]	85.39† [80.24-90.39]	81.75† [75.73-87.81]	84.99† [79.37-90.07]

reports	images	**Pulmonary Infiltrates**				**Pneumothorax**				**Pulmonary Infiltrates**				**Pneumothorax**			
14580	12935	80.64 [78.34-82.77]	82.27 [80.1-84.52]	82.17 [79.89-84.3]	81.91 [79.74-84.1]	72.51 [65.67-78.72]	83.41 [77.33-88.87]	73.93 [66.98-80.54]	84.03 [78.12-89.51]	73.27 [64.78-80.66]	81.27 [74.76-87.17]	79.13 [72.01-85.68]	77.3 [69.02-84.09]	79.07 [69.93-86.71]	90.31 [83.14-95.99]	80.16 [70.87-88.38]	91.85 [85.52-96.24]
7000	6206	78.53 [75.95-80.92]	81.44 [79.27-83.57]	82.61 [80.3-84.66]	81.72 [79.54-83.75]	67.55 [61.36-73.52]	77.16 [70.43-82.94]	77.46 [70.89-83.88]	79.81* [73.91-85.73]	76.25 [67.74-83.74]	79.43 [72.85-86.01]	79.24 [71.98-86.1]	77.83 [70.8-85.19]	71.22 [61.86-80.37]	84.66 [75.76-92.16]	85.2 [77.87-91.58]	88.04* [80.77-93.38]
3500	3096	78.72 [76.29-81.03]	81.16 [78.93-83.19]	82.02 [79.76-84.05]	81.06 [78.75-83.18]	65.82 [59.21-72.24]	78.58 [71.69-84.96]	78.77* [72.65-84.91]	78.5 [72.18-84.87]	78.7 [71.48-85.69]	76.13 [67.63-83.82]	77.51 [70.34-84.5]	75.95 [67.71-83.53]	70.18 [60.41-79.93]	89.16* [81.83-95.09]	87.97* [80.66-93.6]	88.75* [82.05-94.56]
2000	1773	74.08 [71.57-76.59]	81.83* [79.7-83.85]	82.42* [80.18-84.5]	81.56* [79.17-83.7]	68.08 [60.87-74.55]	73.99 [66.74-80.96]	77.43 [71.06-83.56]	76.67 [70.32-82.7]	68.18 [59.74-75.88]	79.35 [72.02-85.38]	77.54 [69.79-84.55]	77.82 [70.55-84.28]	69.88 [58.67-80.63]	79.14 [69.52-88.05]	83.83 [74.67-92.22]	81.14 [72.18-89.56]
1000	877	70.33 [67.69-72.99]	80.88* [78.56-83.14]	83.1* [80.87-85.41]	81.57* [79.43-83.75]	59.91 [52.31-66.89]	77.51* [70.56-84.47]	74.24* [68.18-79.76]	77.57* [70.83-84.59]	63.58 [53.68-72.44]	74.46 [66.34-81.88]	81.29* [73.94-87.35]	75.56 [67.65-83.27]	65.86 [54.82-76.14]	86.27* [77.81-93.22]	85.54* [77.94-91.83]	85.29* [77.52-92.69]
500	450	72.35 [69.38-74.83]	79.07* [76.6-81.5]	80.74* [78.43-83.01]	78.64* [76.09-80.9]	63.85 [57.89-70.3]	73.55 [66.96-79.98]	76.27* [70.41-81.78]	72.82 [66.37-79.6]	68.99 [59.7-77.18]	75.77 [68.01-82.89]	74.76 [67.33-82.43]	72.19 [64.06-79.69]	60.77 [49.45-71.3]	75.28 [65.5-84.48]	84.93* [77.94-91.5]	71.43 [60.69-81.65]

Table 2. Table 2. Area under the receiver operating characteristic curve (AUC) in % observed for the hold-out test set of 2099 images that were labeled by report content and for the hold-out test set of 187 images that were labeled by re-evaluating imaging. The image-based models were trained on report-based labels with four different approaches: solely on gold-labels (M_G), solely on silver-labels (M_S), first with silver, then with gold labels ($M_{S/G}$) and with silver and gold labels together (M_{S+G}). The transformer and image-based models were trained with various numbers (N) of gold-labeled reports and images to investigate the influence of annotation effort on DDSS model performance. For MS, solely silver-labeled images were used generated by the transformer trained with N gold-labels. The highest performances of the models trained with the same number of gold-labels are indicated by bold font for both test sets. Significant difference between the AUCs of M_G and M_S or M_G and M_{S+G} or M_G and $_{MS/G}$ are indicated by * and between the AUCs of the same model (M_G/M_S/M_{S+G}/M_{S+G}) tested on report- or image-based labels with †.

References

1. Huang G, Liu Z, Van Der Maaten L, Weinberger KQ (2017) Densely connected convolutional networks. In proceedings of CVPR 2017 4700-4708
2. Nakayama H, Kubo T, Kamura J, Taniguchi Y, Liang X (2018) doccano: Text Annotation Tool for Human. Available via https://github.com/doccano/doccano. Accessed 28 Jul 2022
3. Nowak S, Biesner D, Layer YC et al (2023) Transformer-based structuring of free-text radiology report databases. Eur Radiol 33:4428-4236. https://doi.org/10.1007/s00330-023-09526-y
